# Access to specialist cancer care: is it equitable?

**DOI:** 10.1038/sj.bjc.6600640

**Published:** 2002-11-12

**Authors:** E Pitchforth, E Russell, M Van der Pol

**Affiliations:** Department of Public Health, University of Aberdeen, Aberdeen, UK; Health Economics Research Unit, University of Aberdeen, Aberdeen, UK

**Keywords:** cancer registration data, inequalities, cancer services, regression analysis, monitoring

## Abstract

The first principle of the Calman–Hine report's recommendations on cancer services was that all patients should have access to a uniformly high quality of care wherever they may live. This study aimed to assess whether the uptake of chemotherapy for colorectal cancer varied by hospital type in Scotland. Hospitals were classified according to cancer specialisation rather than volume of patients. To indicate cancer specialisation, hospitals were classified as ‘cancer centres’, ‘cancer units’ and ‘non-cancer’ hospitals. Colorectal cancer cases were obtained from cancer registrations linked to hospital discharge data for the period January 1992 to December 1996. Multilevel logistic regression was used to model the binary outcome, namely whether or not a patient received chemotherapy within 6 months of first admission to any hospital. The results showed that patients admitted first to a ‘non-cancer’ hospital were less than half as likely to go on to receive chemotherapy as those first admitted to a cancer unit or centre (OR=0.28). This result was not explained by distance between hospital of first admission and nearest cancer centre, nor by increasing age or severity of illness. The study covers the period immediately preceding the introduction of the Calman–Hine report in Scotland and should serve as a baseline for future monitoring of access to specialist care.

*British Journal of Cancer* (2002) **87**, 1221–1226. doi:10.1038/sj.bjc.6600640 www.bjcancer.com

© 2002 Cancer Research UK

## 

The Calman–Hine report in England and Wales in 1995 (Calman and Hine, 1995) and the Scottish version in 1996 (SCCAC, 1996) recommended a new structure for cancer services. This structure was based upon a hierarchy of expertise in cancer care reaching from primary care through cancer units in district hospitals to specialised cancer centres so that ‘care should be provided as close to the patient's home as is compatible with high quality, safe and effective treatment’. The rationale was a growing body of evidence indicating that outcomes in treatment of common and uncommon cancers improve with specialisation (Hakama *et al*, 1989; Gillis and Hole 1996; Selby *et al*, 1996; Hoffman *et al*, 1997; Schrag *et al*, 2000).

Whilst there is a consensus that centralisation of services has unarguable advantages (Campbell *et al*, 1999), specialised centres are more likely to be located in urban areas resulting in geographical inequality of distribution. Studies in the United States, France and Scotland have all shown that patients from rural areas are less likely to be treated in a specialist centre and to have poorer survival rates (Launoy *et al*, 1992; Howard *et al*, 1995; Greenberg *et al*, 1988). Campbell *et al* (2000) indicated that distance from a cancer centre might be a determining factor in survival. A more recent study showed that this is likely to be partly because patients living further from cities and associated cancer centres have more advanced disease at diagnosis (Campbell *et al*, 2001). It is therefore crucial that any increase in physical distance from specialised services does not exacerbate inequalities in uptake of specialist care.

Both reports emphasised the need to monitor the achievement of the proposed equalisation of access and its impact on treatment and survival rates. This study aimed to assess whether the uptake of chemotherapy for colorectal cancer in Scotland varied by type of hospital of first admission. Colorectal cancer was chosen as it is one of the three most common cancers in both sexes in Scotland (ISD, 2001). Although surgery is the single most effective treatment for colorectal cancer, chemotherapy should be considered for both adjuvant and palliative purposes and is considered here as a process indicator of specialist care (SIGN, 1997; Moir, 1999). During the early 1990s, chemotherapy was still relatively new, and was likely to be more sensitive to variation in uptake than it may be now. Mcleod (1999) showed that from 1990 to 1994 in Scotland the onsite provision of chemotherapy at hospital of first admission was significantly positively associated with patients' odds of receiving chemotherapy. A further aim was to update the analysis to the period immediately before the introduction of clinical guidelines for colorectal cancer management (SIGN, 1997) and the Calman–Hine recommendations so that it could serve as a baseline study against which change could be monitored.

## MATERIALS AND METHODS

Regression analysis was used to examine the effect of degree of hospital specialisation on uptake of chemotherapy. The regression model used a three-level hierarchy of patients nested within areas within hospitals. The dependent variable was whether a patient received chemotherapy within 6 months of first admission. Comparison with the study for 1990–94 was made using descriptive statistics and adjusted odds ratios with 95% confidence intervals. The definitions of the variables are given below.

### Patients

The analysis was based on the same data set for 1992–1996 as used by Austin and Russell (2002). Scottish cancer registration records for all patients aged over 17 diagnosed between 1st January 1992 and 31st December 1996 (*n*=15299) formed the study population. Their data were linked by computerised probability linking by the Information and Statistics Division (ISD) Scotland to individual episodes of care collected through the Scottish Morbidity Record inpatient and day case form (SMR 01). SMR 01 details were selected if they contained a diagnosis of colorectal cancer and were within the time frame of 1 year pre-registration to 1-year post registration.

To answer this specific research question and to fulfil the requirements of multilevel modelling a number of exclusions were necessary. To permit comparison with the previous study by Mcleod (1999), several of the same definitions were used. Patients with no recorded hospital admissions were excluded. Consistent with Mcleod (1999), 6 months from date of first hospital admission was taken as the period in which uptake of chemotherapy was recorded, as almost 90% of patients who received chemotherapy did so within this period. Patients admitted after the 30th June 1996 were therefore excluded (*n*=1517). Of the remaining 11 728 cases, those with no recorded deprivation category (Depcat) (Carstairs and Morris, 1991) or postcode, patients who died on day of diagnosis and those treated in an English hospital were excluded (214).

For the regression analysis there must be a sufficient number of cases within each level and category. Only 47 out of 4123 patients aged 75 and more received chemotherapy and therefore all patients aged 75 and over had to be excluded from the analysis. Similarly, hospitals that admitted fewer than five colorectal patients in the 5-year period were excluded (77 hospitals, 180 patients). The final data set used in the multilevel analysis contained 7303 cases.

### Level of hospital specialisation

Three levels of cancer specialisation were defined for the 48 Scottish hospitals that treated five or more colorectal cancer patients in the 5 year period: cancer centre, cancer unit and ‘non-cancer’ hospital. The Calman–Hine proposals identified the five cities of Aberdeen, Dundee, Edinburgh, Glasgow and Inverness as cancer centres and described the role of centres (highly specialised) and cancer units (commoner cancers), but no formal administrative classification yet exists of which hospitals in Scotland are cancer centres or units. After consultation with the Cancer Surveillance Unit in ISD and other cancer experts, nine hospitals within the five cities were identified as ‘cancer centres’. Using the Health Services Cost Books (National Health Service in Scotland ISD, 1996), hospitals coded as 01 (major teaching hospital), 02 (general hospitals), 11 and 12 (mixed speciality hospital with or without maternity) but which were not already cancer centres were judged to be ‘cancer units’ (total 24 hospitals). The remaining 15 hospitals were classed as ‘non-cancer’ hospitals. The expected journey of care for colorectal cancer patients, as one of the commoner cancers, was that they would usually be referred on to a cancer unit for chemotherapy but might also be referred to a centre. Hospital of first admission was therefore used for the analysis of hospital effect, and the process of onward referral was treated as integral to whether or not chemotherapy was given.

### Area level variables

Patients were grouped into geographical areas using postcode sectors. The 7303 cases included in the analyses were resident in 848 areas and admitted to one of 48 hospitals. Depcat was used as an area level deprivation measure (Carstairs and Morris 1991). Depcat 1 was categorised as ‘most affluent’, Depcat 2 and 3 as ‘mid-affluent’, Depcat 4 and 5 ‘mid-deprived’ and Depcat 6 and 7 as ‘most-deprived’ quartile. Rurality was based on population density per hectare (Mcleod, 1999). Postcode sectors with density <6 were termed ‘rural’ whilst those ⩾6 were ‘urban’.

### Patient level variables

Age and sex were taken from the linked data. Co-morbidity was coded as ‘none’, ‘one’, or ‘two or more’ admissions from any of the relevant conditions (Austin and Russell 2002). Patient transfer and waiting list cases were grouped together as ‘elective’ and all others as ‘emergency’. Dukes' staging, which would have been the severity indicator of choice, was not available in cancer registration data at the time. Instead, ‘death within the first 6 months’ was used as an indication of severity of illness (Mcleod, 1999). Since the date of death was not included in the linked data, days from diagnosis to death was used. *Post hoc* analysis of Dukes' staging from the 1997 cancer registration data (which contained staging for the first time) was used to assess the likely percentages of patients with different stages of cancer (Austin and Russell, 2002).

### Clinical data

SIGN guidelines currently recommend adjuvant chemotherapy for patients with stage C colorectal cancer, entry into randomised trials for Dukes' stage B, and palliative chemotherapy for all patients with advanced or metastatic cancer. It is likely that in the early 1990s Dukes' C was the only clear indication for chemotherapy. The coding of chemotherapy (X35.2) in the cancer registration data does not distinguish between adjuvant and palliative, and therefore the presence or absence of surgery was included in an attempt to make this distinction. Definitive potentially curative surgery was defined following consultations with a colorectal surgeon and reference to the published literature (Austin and Russell, 2002).

### Regression analysis

In the regression model the data are hierarchical, in that patients are nested within areas within hospitals. This is done to avoid overestimation of the statistical significance of explanatory variables that may arise because the observations are not independent of one another. The technique allows variation which occurs at the higher levels (i.e. variation amongst postcode sectors or hospitals) to be analysed separately from variation at the level of the patient. Because the dependent variable was binary, logistic regression was used to model the binary outcome, i.e. whether or not a patient received chemotherapy. Models were estimated using restricted iterative generalised least squares (Goldstein, 1995). The effect of each variable upon the likelihood of receiving chemotherapy was compared by conversion from a coefficient to odds ratio (OR).

From the results of the regression analysis, statistical significance was tested by dividing each estimate by its standard error, resulting in a *t*-statistic. If the *t*-statistic was in excess of ±1.96, the estimate was judged significantly different from zero at the 0.05 level.

## RESULTS

Austin and Russell (2002) provide descriptive statistics from the larger data set.

Before making the necessary exclusions, the full data set contained 15 299 cases. Of the 13 245 cases with at least one hospital admission, 10.7% (1422) received chemotherapy. Of these patients, 76.2% (1083) received definitive surgery within one year, indicating that chemotherapy was provided on both adjuvant and palliative bases.

The characteristics of the 7303 patients aged less than 75 years are listed in Table 1

**Table 1:**
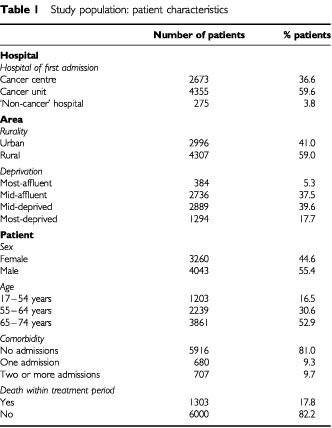
Study population: patient characteristics

. A total of 13.7% (999) patients received chemotherapy within 6 months of first admission. The majority of all cases, 70.3% (5133), presented as elective admissions. As would be expected from the nature of colorectal cancer most patients, 83.5% (6100), were over 54 years of age. Using co-morbidity as defined, 81% (5916) had no admissions for any of the relevant conditions, 9.3% (680) had one admission and 9.7% (707) had two or more admissions. Six thousand (82.2%) lived beyond the defined treatment period of 6 months from date of diagnosis. Of those who died within the 6 month period (*n*=1303), 8.7% died within 14 days from date of diagnosis and 29.5% within 90 days. Over half of the patients were classified as rural (4307, 59%).

Only 3.8% (275) of patients were admitted to a ‘non-cancer’ hospital; 59.6% (4355) were admitted to a cancer unit and 36.6% (2673) to a cancer centre.

Before adjusting for the patient, area and hospital characteristics there was significant variation between hospitals in the percentage of patients receiving chemotherapy. The variation between areas was very small and non-significant. Consequently, area was removed as a level and the remaining analysis was carried out using a two-level model of hospital and patient. After adjusting for the explanatory variables in the two-level model, variation between hospitals remained significant. The effects of the patient, area and hospital characteristics upon the likelihood of receiving chemotherapy are shown in Table 2

**Table 2:**
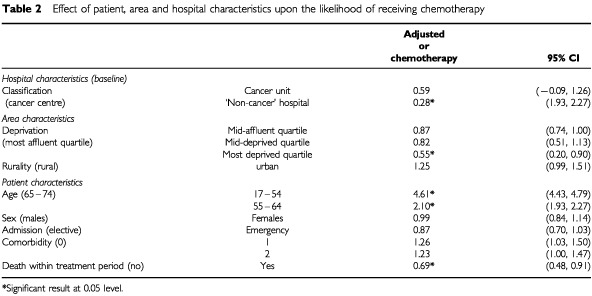
Effect of patient, area and hospital characteristics upon the likelihood of receiving chemotherapy

.

### Patient effects

There was a large effect with increasing age despite the exclusion of patients over 75 years. Patients aged 17–54 years were over four and a half times more likely to receive chemotherapy than those aged 65–74 years (OR=4.61, 95% CI 4.43, 4.79). There was no significant difference between males and females. Patients admitted as emergencies were less likely to receive chemotherapy but the difference was not significant. Co-morbidity did not have a significant effect but patients who died within 6 months were significantly less likely to receive chemotherapy.

Recognising the limitations of co-morbidity and death within 6 months as indicators for disease severity, a *post hoc* analysis was conducted on the comparable set of 1997 data, which for the first time contained Dukes' staging (Austin and Russell, 2002). There was no significant variation in staging across age groups, indicating that the decreased likelihood of receiving chemotherapy in older patients was not directly explained by increased cancer severity. Moreover the percentage with unknown staging did not increase significantly with age below the age bands of 75 and above. However, the uptake of chemotherapy did. Taking only Dukes' stage C as an indicator for chemotherapy (SIGN, 1997), the 1997 data showed that 27.5% of patients all patients were Dukes' C and 35.3% of these patients received chemotherapy. Looking at the uptake of chemotherapy among Dukes' C patients within each age group, 82.1% of those aged 17–54 years, 61.6% of those aged 55–64 years, 36.6% of those aged 65–74 years and only 6.6% of those aged over 75 years with Dukes' C received chemotherapy.

### Area effects

There was evidence of a deprivation effect as those in the most deprived quartile were just over half as likely to receive chemotherapy as those in the most affluent quartile. Rurality did not have a statistically significant effect.

### Hospital effects

There was a significant effect with hospital classification. Compared to a cancer centre, patients admitted first to a cancer unit or ‘non-cancer’ hospital were less likely to receive chemotherapy although this was significant only for those admitted to a ‘non-cancer’ hospital. A *post hoc* analysis of the effect of distance was included to explore whether this effect was explained by increasing distance from hospital of first admission to the nearest cancer unit or centre. Distance was grouped as <95 km and ⩾95 km (straight line distance) and an interaction term with ‘non-cancer’ hospital was included in the model. This interaction was not statistically significant indicating that distance did not appear to be a determining factor.

### Onward referral from non-cancer hospital

There was expected to be a close correlation between uptake of chemotherapy by first admissions to a non-cancer hospital and whether or not the patients were referred on to a cancer unit or centre. Of the 275 patients (3.8%) admitted to a ‘non-cancer’ hospital, 201 (73%) had no further admissions for colorectal cancer. Compared to those referred on, those not referred on tended to be more deprived (56.2%) (χ^2^=10.539, *P*-value=0.014), after merging the deprivation quartiles into ‘most affluent’ and ‘most deprived’ (Table 3

**Table 3:**
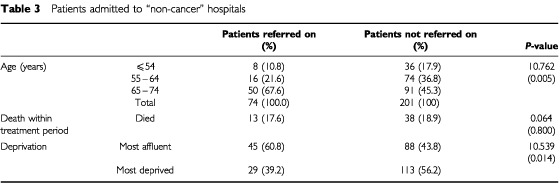
Patients admitted to “non-cancer” hospitals

). Those not referred on also tended to be younger with a lower proportion in the 65–74 year age group (45.3%) (χ^2^=10.762, *P*-value=0.005). Therefore the difference between referrals and non-referral is not explained by increasing age although deprivation may be a factor. A greater proportion of those not referred on died within the treatment period suggesting greater disease severity as a reason for non-referral but this difference was not significant. We are unable to explain the varied associations with chemotherapy found in this subgroup of patients.

### Time trends

To give some indication of variation over time a direct comparison was made to McLeod's (1999) study based on the 1990–1994 cancer registration data (Table 4

**Table 4:**
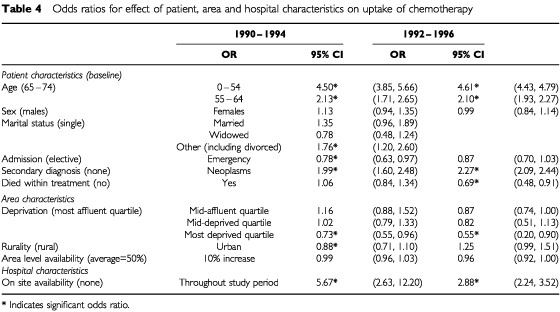
Odds ratios for effect of patient, area and hospital characteristics on uptake of chemotherapy

). In this analysis hospitals were classified according to onsite availability of chemotherapy (>5 recorded episodes of chemotherapy) and secondary diagnosis was used as an indicator of comorbidity. The patient characteristics were very similar in the analyses of the 1990–1994 and 1992–1996 cancer registration data. The number of hospitals included (59) was greater in 1990–1994 but a similar proportion of patients was first admitted to a hospital with onsite availability of chemotherapy.

For all ages, the rate of chemotherapy uptake increased by 2.7% from the 1990–1994 study, from 8% to 10.7%. In 1990–1994, as in this study, a significant negative effect was found for increasing age and deprivation, and a positive effect of on site availability and co-existent neoplasms. As there is a two year overlap between the two studies similarities would be expected and their existence supports the findings of both studies. Whilst the trend of significant results is largely similar, there are some interesting differences in the size of effect between the two studies. The effect of a secondary diagnosis of neoplasm increased from OR 1.99 to OR 2.27, while there was a slight fall in the OR for deprivation (from 0.73–0.55). The largest difference between the two studies was in the size of effect of on site availability at hospital of first admission. In 1990–1994 patients who were first admitted to a hospital with on site availability were almost six times more likely to receive chemotherapy than those admitted to a hospital with no onsite availability (OR=5.67, CI 2.63–12.20). The effect was still significant in 1992–1996 but was greatly reduced (OR=2.88, CI 2.24–3.52). Patients from 1992–1996 were still almost three times less likely to receive chemotherapy if they were not first admitted to a hospital with onsite chemotherapy. This follows the establishment of the Scottish Cancer Therapy Network in 1993 and may reflect more widespread appreciation of the benefits of specialist care and the role of non-surgical techniques in the treatment of colorectal cancer.

## DISCUSSION

The leading principle behind the Calman–Hine recommendations is that all patients should have access to a uniformly high quality of care in the community or hospital wherever they may live to ensure maximum possible cure rates and best quality of life (Calman and Hine, 1995). Since the time of the study data, the emphasis has become more specifically on access to multidisciplinary teams within hospitals rather than simply to specialist hospitals. However, the labelling of specialists and teams is still a matter of judgement, and our classification of hospitals was based on knowledge of the health service and not on a set standard for level of specialisation. Therefore, some hospitals may have been inappropriately classified. The results are encouraging however as they discriminate well among the three types of hospital. Discussion with oncologists has confirmed that almost all the cancer units had weekly visits from oncologists but this was not the case for the non-cancer-hospitals. Thus the level of specialisation available by hospital was relatively homogenous within the three categories and discrete among them. It was not possible to analyse for differences between individual clinicians' practice within units or centres, but the classification enabled a focus on whether or not non-cancer hospitals were playing their part in onward referral to specialist care as judged by type of hospital and uptake of chemotherapy.

This analysis of routine cancer registration and hospital discharge data has shown that patients admitted to a ‘non-cancer’ hospital were less likely to receive chemotherapy than those admitted to a cancer unit or centre. At 3.8% of those aged less than 75, it is a relatively small proportion of all cancer patients to whom this applies. However it involves 15 of the 48 hospitals in Scotland that treat colorectal cancer patients and may indicate an important problem associated with increased specialisation. Older patients were also shown to be less likely to receive chemotherapy. The low numbers of patients aged 75 or more who received chemotherapy and evidence from Austin and Russell (2002) that shows older age as a statistically significant negative predictor for receiving chemotherapy suggest that the effect may well be greater in those patients over 74 years of age. It is therefore a possible inequity that does not sit well with the principles of equal access to specialist care embodied in the Calman–Hine report (Calman and Hine, 1995).

As in the study by Austin and Russell (2002), reasons for the age and hospital effect would be largely speculative. More in-depth, qualitative studies are needed to understand why older patients and patients admitted to a ‘non-cancer’ hospital may be less likely to go on to receive specialist cancer care. Previous studies have shown patients living more remote from cities and cancer centres have more advanced cancer at diagnosis and poorer outcomes (Campbell 2000, 2001). This must not be compounded by an inequity of access to specialist care. This study has provided a possible method for monitoring access, a system of hospital classification and a baseline for future analysis that will be helped by the inclusion of Dukes' staging in future cancer registration data.
